# The self-care process of community-dwelling older adults during the COVID-19 pandemic

**DOI:** 10.1590/0034-7167-2022-0644

**Published:** 2023-03-06

**Authors:** Larissa Padoin Lopes, Daniela Bulcão Santi, Francielle Renata Danielli Martins Marques, Maria Aparecida Salci, Lígia Carreira, Vanessa Denardi Antoniassi Baldissera

**Affiliations:** I Universidade Estadual de Londrina. Londrina, Paraná, Brazil; II Instituto Federal Catarinense. Blumenau, Santa Catarina, Brazil; III Universidade Estadual de Maringá. Maringá, Paraná, Brazil

**Keywords:** Self Care, COVID-19, Aged, Primary Prevention, Public Health Nursing., Autocuidado, COVID-19, Anciano, Prevención de Enfermedades, Enfermería en Salud Pública., Autocuidado, COVID-19, Idoso, Prevenção de Doenças, Enfermagem em Saúde Pública.

## Abstract

**Objectives:**

to understand the self-care process of community-dwelling older adults during the COVID-19 pandemic.

**Methods:**

this is an explanatory study with a qualitative approach based on the constructivist Grounded Theory, carried out with 18 community-dwelling older adults. Data collection took place through interviews and content was analyzed through initial and focused coding.

**Results:**

two categories were obtained: “Building connections to support self-care practices” and “Living with the risk group stigma”. From their interaction, the phenomenon “Performing self-care in old age during the COVID-19 pandemic” emerged.

**Final Considerations:**

it was possible to identify how older adults’ experiences curing the COVID-19 pandemic had repercussions on their self-care process, being influenced by factors such as information about the disease and the impacts of risk group stigmas.

## RESUMO

**Objetivos:**

compreender o processo de autocuidado dos idosos da comunidade no contexto da pandemia de COVID-19.

**Métodos:**

trata-se de um estudo explicativo de abordagem qualitativa, com referencial da Teoria Fundamentada nos Dados construtivista, realizada com 18 idosos comunitários. A coleta de dados ocorreu através de entrevistas, e o conteúdo foi analisado por meio da codificação inicial e focalizada.

**Resultados:**

foram obtidas duas categorias: “Estabelecendo conexões para apoiar práticas de autocuidado” e “Convivendo com o estigma de grupo de risco”. A partir da interação dessas, emergiu o fenômeno “Exercendo o autocuidado na terceira idade no contexto da pandemia de COVID-19”.

**Considerações Finais:**

foi possível identificar como as vivências dos idosos no período de pandemia de COVID-19 repercutiram no seu processo de autocuidado, sendo influenciados por fatores, como informações sobre a doença e os impactos dos estigmas do grupo risco.

## RESUMEN

**Objetivos:**

comprender el proceso de autocuidado de los ancianos en la comunidad en el contexto de la pandemia de COVID-19.

**Métodos:**

se trata de un estudio explicativo con enfoque cualitativo basado en la Teoría Fundamentada en Datos constructivista, realizado con 18 ancianos residentes en la comunidad. La recolección de datos se realizó a través de entrevistas y el contenido se analizó a través de una codificación inicial y focalizada.

**Resultados:**

se obtuvieron dos categorías: “Establecer vínculos para apoyar prácticas de autocuidado” y “Convivir con el estigma de grupo de riesgo”. De su interacción surgió el fenómeno “Ejercer el autocuidado en la vejez en el contexto de la pandemia del COVID-19”.

**Consideraciones Finales:**

fue posible identificar cómo las vivencias de los adultos mayores durante el período de la pandemia de COVID-19 repercutieron en su proceso de autocuidado, siendo influenciados por factores como la información sobre la enfermedad y los impactos de los estigmas del grupo de riesgo.

## INTRODUCTION

Coronavirus (COVID-19) disease is an infectious disease characterized by the potential severe acute respiratory condition, which began to spread in November 2019, in the city of Wuhan, China. Due to the rapid spread of the disease in hundreds of countries, the World Health Organization (WHO) declared a pandemic situation on March 11, 2020, and in Brazil, until February 2022, 26,599,593 cases had been confirmed and 632,621 deaths caused by the disease^(1-2)^.

With the large scale of confirmed cases and deaths, the risk of the disease developing severe forms was evidenced, especially in the risk group, such as older adults and immunosuppressed, among others^(2)^. It is known that in the world, until 2020, the population of older adults was composed of about 1.1 billion people and, in Brazil, about 29.9 million^(3)^. This population was strongly affected by the disease, since, in Brazil alone, from February to September 2020, COVID-19 reached about 66% of people aged 70 years or older and that 76% of deaths related to this disease occurred in the population aged 60 years or older^(4)^.

Older adults, in turn, also suffer stigmas that surround this phase of life and that were reinforced in the pandemic scenario, mainly because they have greater health vulnerability and develop several comorbidities, mainly non-communicable chronic diseases, such as high blood pressure, diabetes mellitus, among others^(5-6)^.

It is evident that the COVID-19 pandemic directly impacted older adults’ quality of daily life. Faced with the moment experienced, which imposed a change in routine due to the need for social isolation, one of the main ways to prevent contagion, it is presumed that several conflicts and feelings were experienced by this population as well as attitudes and behaviors as ways of coping. Social isolation requires other measures, such as distancing from family members who live in the same household, use of communication technologies, hand and food hygiene measures, use of a mask, among others^(3,7)^.

Thus, the Primary Health Care (PHC) role as one of the main points of the care network in combating and preventing the COVID-19 pandemic is emphasized. PHC performs the functions of health protection, prevention and control of infectious diseases, individual and family follow-up and the population’s monitoring and health education, thus having operational capacity to detect and treat mild and moderate cases in a timely manner^(8-9)^.

Therefore, older adults demanded, together with PHC, the development of self-care strategies and actions to prevent the disease, as the main source of the health system, which leads to a decrease in hospital occupancy rates, the collapse of the health system and the mortality rate of this population. It is also emphasized that lack of compliance with preventive behavior leads to ineffective control and the rapid spread of the disease, characterizing the population’s self-care process as a public health emergency^(8,10)^.

Due to the pandemic scenario exceptionality and field research literature scarcity that addresses older adults’ perspectives and behaviors for disease prevention, it becomes necessary to build knowledge that reflects the experiences of this population in this period, in order to support effective health strategies in these situations or their consequences.

## OBJECTIVES

To understand the self-care process of community-dwelling older adults during the COVID-19 pandemic.

## METHODS

### Ethical aspects

All ethical precepts guided by Resolutions 466/2012 and 510/2016 of the Brazilian National Health Council were respected. Participants were informed about the research and signed the Informed Consent Form in two copies, keeping a copy. The research is part of a more comprehensive study and has the approval by the Standing Committee on Ethics in Research with Human Beings. To ensure participant anonymity, older adults’ statements were coded with the letter “P”, to refer to the term “Participant”, followed by Arabic numbers that corresponded to the order in which the interviews were carried out with each group.

### Study design

This is an explanatory study with a qualitative approach, anchored in the constructivist Grounded Theory (GT) theoretical-methodological framework. The GT aims to understand individuals’ experiences and interactions in a given social context, thus demonstrating the strategies developed in the face of the phenomena experienced^(11)^.

The COREQ (Consolidated criteria for reporting qualitative research) protocol was used in order to improve the presentation of the results of this research.

### Study setting

The research was carried out in a Basic Health Unit (BHU), which is part of the Older Adults Health Care Network in the city of Maringá, Paraná. This BHU was the site of an extension project developed by the researchers since 2016, which had face-to-face practices with older adults interrupted during the pandemic.

### Participants and data source

Eighteen older adults participated in this study. Individuals being 60 years old or older and being duly registered at the BHU were included. Participants with altered cognitive function according to the Mini Mental State Examination (MMSE) were excluded^(12)^. First, the search for participants took place through the analysis of medical records of older adults assisted by the project in the unit.

Interview analysis, initially carried out with eight older adults with no history of diagnosis by COVID-19, suggested the hypothesis that the COVID-19 pandemic negatively impacted older adults’ routine and autonomy, although with a relevant manifestation of resilience among them. This perspective added the following questions: what would be the self-care perspectives experienced by older adults who were diagnosed with COVID-19?

Therefore, it was considered relevant to include a second sample group of older adults, adding the criterion of history of positive diagnosis of COVID-19. After a new analysis of medical records and contacts with the unit professionals, 10 more older adults were interviewed.

### Data collection and organization

Data collection took place from September to October 2021 through semi-structured individual interviews, using a questionnaire prepared and validated by judges with expertise in the subject^(13)^. The questions covered sociodemographic characteristics, such as sex, age, marital status, social housing status, education and per capita family income, in addition to guiding questions related to experiences and perceptions of self-care during the COVID-19 pandemic.

The interviews were conducted by the main researcher, who has considerable experience in assisting older adults in Primary Care through the developed extension project. Contact with older adults was made, first, from home visits, and considering the difficulty in face-to-face access, interviews were conducted via telephone. It should be noted that in these cases the Brazilian Telephone Mini-Mental State Examination (Braztel-MMSE)^(14)^ was used, maintaining the research methodological rigor. On these occasions, previously, the researcher presented the research objective and data collection method.

The interviews lasted about 12 minutes and were audio-recorded and transcribed in full, being organized in a Microsoft Excel 2018^®^ spreadsheet. Data collection ended due to theoretical saturation, when the interviews did not provide other reflections about the categories built in the process or adjacent^(15)^.

### Data analysis

Data analysis was performed in two coding steps: initial and focused^(11)^. In the initial coding, data were fragmented and later analyzed line by line in order to conceptualize the findings expressed by research participants, transforming them into codes. In the focused coding, more targeted, selective and conceptual codes were elaborated, synthesizing and explaining the largest segments of data, which led to obtaining the phenomenon or central category of research^(11)^. To organize and categorize the data, the software MAXQDA^®^ was used for the Windows operating system, which assists in qualitative data analysis, from the coding tool in vivo^(16)^.

The analysis process was shared among the study researchers and 89 codes were elaborated in the analysis process, whose relationships allowed building the study phenomenon.

## RESULTS

As for the characterization of the 18 older adults who participated in this research, it is believed that they were between 61 and 82 years old, most of whom were female (14). Regarding marital status, the following were declared: married (8), widowed (5), single (2), common-law marriage (2) and divorced (1). However, only two older adults lived alone. Regarding education, they had: incomplete elementary school (13), complete high school (3), higher education (1) and no literacy (1). All participants reported family income greater than two minimum wages.

With regard to preexisting comorbidities, 15 older adults reported having diseases such as diabetes, hypertension, hyperthyroidism, bronchitis, depression and anxiety. It is also noteworthy that, during the research period, all older adults had been vaccinated with both doses of COVID-19 vaccine.

From the process of systematic data analysis and integration, the phenomenon of a study entitled “Performing self-care in old age during the COVID-19 pandemic” emerged, supported by two interrelated categories described below.

### Building connections to support self-care practices

Community-dwelling older adults needed to change their routine during the pandemic, including new habits for self-care and disease prevention. Thus, several sources of information mediated this process, and the reference to health professionals is relevant.

[The nurse] *said that it is to prevent, not to walk around without a mask, to wash your hands.* (P2)

*When we go there* [at the BHU] *the girls always explain. They say that it is not to keep crowds, to be careful, to keep away, to wear a mask. The care they need.* (P5)

*A lot of people gave me this recommendation, the ones I was already doing: gel alcohol, distancing, mask.* (P17)

*I went to the south zone, went to the physician, went to the nurse. They told us to wear a mask, right?* (P11)

Some older adults did not report receiving guidance from health professionals, but also highlighted that they provided care and emphasized the importance of this. Social isolation was one of the main measures recommended to prevent the spread of the disease. Thus, technological support has become an important means for access to information and guidance on this, until then, new disease. Such technologies used by older adults included television, radio, cell phone and internet.

*I didn’t receive any guidance from professionals, always at the beginning, the newspaper advised how we should do it. In that group that we went to ‘Feeling good about life’, there were many teachings for older adults to avoid getting hurt, falling. But about COVID, no* [...] *ah it’s important, my daughter, the best thing we do is prevention.* (P16)

*We saw it on television, the care, like, that we’ve been taking, it was all on television.* (P15)

*In addition to the health center? The media, right. That, TV, cell phone* [...]. (P13)

*We hear it on TV and YouTube^®^ too.* (P14)

It is inferred that prevention practices carried out by older adults are anchored in the guidelines received by the media. Among these practices, vaccination, hand hygiene, mask use and social distancing stand out.

*I use my normal mask, wash my hands, when I need to go to the market, I apply the alcohol gel.* (P1)

*I use alcohol, I don’t leave the house without wearing a mask, everywhere I go, I wear a mask* [...] *we still don’t go to church.* (P10)

*We only went out to go to the market, buy the things we needed and came back home, always being careful, we didn’t go to the house of friends, relatives, anyone.* (P18)

*Avoid accumulating people, using alcohol gel and mask.* (P9)

In addition to the measures mentioned above, some older adults reported performing practices not recommended by professionals or responsible health bodies. Using technology as a means to access information favored contact with erroneous recommendations (fake news), which had great circulation in this period. It is highlighted as relevant in this research that reports of such practices not officially recommended by regulatory bodies emerged only in the group of older adults who had a positive diagnosis for COVID-19.

*Then I made a mug of garlic tea with lemon and ginger, I drank three coffee mugs of this tea, you know? I took three with a flu pill, in the morning, at noon, at night, for me it was the flu.* (P16)

*My husband didn’t take it because he had taken Ivermectin* ^®^ *, and on the day I was supposed to take it, I didn’t take it, that’s why I took it, but as I took the Ivermectin* ^®^ *, I quickly got better* [...] *the physician gave antibiotics, syrup, and I took it, but I took Ivermectin* ^®^ *on my own.* (P17)

On the other hand, the older adults who caught the disease report having continued with the same preventive measures taken before the positive diagnosis; however, some made them more flexible and others intensified them.

*The same thing, the same way I did it, exactly the same. I haven’t changed at all.* (P16)

*I continue to take the same care, not like I used to, when the pandemic was at its peak, now I am less than that time*. (P17)

*No, I changed even more, if I already took care of myself, if I took care of myself, now I take care much more, you know.* (P13)

*The same thing we were doing, more often, because we get scared, right?* [...]. (P12)

### Living with the risk group stigma

Since the onset of the pandemic, older adults have been considered one of the most vulnerable groups to the disease due to several factors, and this has had repercussions on their beliefs and self-care practices. It was possible to apprehend that such practices were permeated by the fear of contracting the disease, its complications, as well as the possibility of reinfection.

*Ah, because we are afraid of catching it* [...] *we are afraid, we prevent ourselves because we are afraid.* (P7)

*Jesus, it was very sad, we are very afraid, yes, I was very afraid of getting it, very afraid* […] *this is the reason, the reason is we are afraid of being hospitalized, we are afraid of, God forbid, dying, the suffering of getting it, avoiding suffering, this motivates me to take the vaccine.* (P13)

[...] *if I don’t have it, I won’t get sick, I won’t be a problem and I won’t pass it on to others.* (P5)

[...] *because, if I catch it, I die.* (P10)

It was also assimilated that older adults who had their activities to maintain daily life and leisure before the pandemic stopped doing them to stay at home, maintaining social distance. Thus, older participants explained that this process had consequences that had a strong impact on them, since it implied abstaining from important situations for health and autonomy, such as physical activity, financial control, contact with friends, among others.

*Yeah, because I went out, I went after my money, I did my shopping, we saw something different, we bought it, we went to friends and we don’t go anymore* [...]. *So, you stopped all that, what do you want, you get irritated, you get nervous. I get nervous.* (P3)

*So, friendships, relationships changed a lot, like from friends, dear people, even people from the church.* (P6)

*Ah, it’s just that we always went down there in the hall, gymnastics wasn’t there anymore, walking wasn’t going anymore, meetings too. It’s all changed.* (P7)

Furthermore, specifically with regard to the mental health of these older adults, repercussions can be verified in the statements that present feelings such as discouragement and loneliness, triggered by the abrupt and involuntary interruption of their daily activities.

*So, no leisure at all, because we didn’t go out anywhere.* (P16)

*I stopped, my passion was recycling.* (P4)

*I found it very difficult, you know, because I only have one grandson and I live far away.* (P8)

*I was excited, now the physiotherapy is back and I don’t feel like going out anymore* […] *but I know it’s up to us not to go out, we don’t have the mood for anything.* (P3)

[...] *you can see that we pass by people and people are afraid to pass close to us.* (P6)

**Figure 1:**
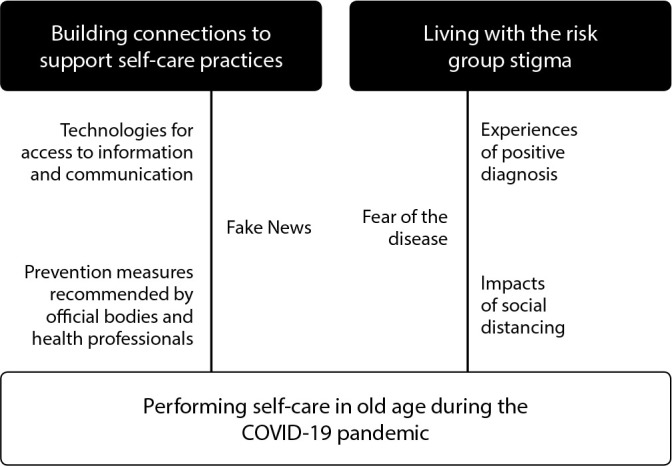
Representative diagram of the interaction of categories with the phenomenon, Brazil, 2022

From articulation of categories explained above and their respective codes, one can observe community-dwelling older adults’ experiences during the COVID-19 pandemic and their self-care elaboration processes. Therefore, for a better understanding of the interaction between category and phenomenon, a representative diagram was created (Figure 1).

## DISCUSSION

It was possible to understand the self-care process of older adults during the COVID-19 pandemic period, noting that this occurred surrounded by the influence of various sources of information and negative feelings experienced by the social stigma of older adults as a risk group.

Older adults reported that they received guidance from health professionals on how to prevent COVID-19. The main guidelines were related to physical contact and hygiene measures that are in line with those recommended by official health bodies, such as hand washing, mask use, alcohol gel and social distancing^(17-18)^. This demonstrates a shift in the period of the COVID-19 pandemic to a normative and prescriptive health care for older adults, which is necessary, although other aspects related to integral health also needed to be elaborated.

PHC professionals’ relevance is highlighted, since they play an essential role in the process of supporting the entire population’s self-care, with emphasis on older adults, being one of the pillars of the public health system. In order to cope with public health emergencies, the link between health team and user, care comprehensiveness, territory knowledge and monitoring of vulnerable families as fundamental PHC actions that enhance and strengthen health promotion and disease prevention as well as correct orientation of the population about the disease stand out^(19-20)^.

With the intention of building self-care strategies, older adults sought information about the disease and how to prevent it. Thus, Information and Communication Technologies (ICT) stand out as the main means used, which include radio, cell phones, television and the internet. It should be noted that ICT are used not only to disseminate information, but, during the COVID-19 pandemic, they were a support to establish communication between people, as well as to control suspected and confirmed cases, systematizing data between states and countries and fight the disease^(21-22)^.

The ICT used by older adults in the research converge with the scientific literature that ratifies technology use as a resource for searching for information by the population worldwide. A study carried out in Italy with 4,260 citizens showed that the most used sources of information were social media, official websites and traditional media, such as television and radio programs, also associating that women over 70 years of age are the ones who use these most sources of information and rely on them^(23)^.

From the various information obtained about the disease, it can be observed that older adults performed self-care practices related to preventive methods that are effective and recommended in the fight against COVID-19, with increased frequency of hand washing, use of alcohol gel several times a day, washing food and wearing a mask^(24-25)^. Social distancing was a relevant measure, as it has significant effects on the speed of spread of the disease; however, this greatly affected older adults, especially with regard to activities of daily living (work, going to the bank, church), family contact and access to health services^(26)^.

However, some practices performed by older adults without scientific basis were observed, anchored in the large circulation of erroneous information in the media, also called fake news. Studies have shown that, during the COVID-19 pandemic, the phenomenon called “Infodemic” occurred, which is the excess of information on a given topic, which generated the spread of false information about the disease in large numbers, influencing the population’s behavior^(27-28)^.

Research shows that the false news circulating on digital platforms has some aspects: some are linked to the disease origin, relating it to conspiracy theories fueled by speeches by political leaders. Another aspect that fake news brings is pseudoscientific health therapies, indicating ineffective prevention measures, alternative or homemade remedies, such as drinking hot drinks to eliminate the virus, indicating vaccination against pneumonia and malaria as effective, among others^(28-29)^.

In line with the findings of this research, it is observed that false information influences self-medication, and in Brazil the dissemination of the so-called “COVID kit” or “early treatment”, composed of medications with antimalarial and antiparasitic indications and vitamin supplements, can have serious adverse effects, especially with regard to older adults^(30-32)^.

In this context of imminent risk of the disease and its still unknown repercussions, older adults were highlighted. This stems from the characteristics of this population, especially with regard to senility, which adds pathological conditions to the aging process, making older adults more vulnerable. It should be noted that, in the United States, the COVID-19 mortality rate in the population aged 70 years or older represented 80% of cases^(33)^.

The effects of the disease on older adults’ health highlighted this group, relating it to the term “risk group”^(2)^. This determination can also reinforce other labels of old age and “old phobic” practices imposed by society^(34-35)^ and that affect older adults’ self-care practices, corroborating actions of social exclusion and strengthening the generalized idea of fragile older adults, without autonomy in their lives, who must be protected by family and society. Moreover, stigmas generated during the COVID-19 pandemic period may influence risk behaviors by the population that is not determined to be a risk group^(34-35)^.

The fear of contracting the disease surrounded not only older adults, but it possibly occurred in an amplified way for them, since they lived with the stigma of being part of the disease risk group and this can have serious consequences for this population, directly affecting their health process^(36-37)^.

Older adults show that they have, in addition to fear, other negative experiences, such as discouragement and loneliness, demonstrating how the COVID-19 pandemic directly impacted older adults’ mental health. It is estimated that about 192 million people over the age of 60 have some level of mental disorder, a prevalence that increased during the confinement period^(33)^. Mental health has a direct impact on the risk of death and on the self-care process of various comorbidities in people aged 75 and over, causing moderate to severe repercussions on older adults who lived through the COVID-19 pandemic^(35)^.

The high amount of information on the internet about the disease can contribute to this impact on mental health. A study carried out with Chinese adults showed that 82% of this population had depression, anxiety and other feelings associated with news found on social networks^(38)^.

Another factor, social isolation, may also have an inherent contribution, since many older adults find themselves alone in their homes. The family arrangement of Brazilian older adults can be constituted in different ways; however, this arrangement often had to be changed due to the need to reorganize the routine and family dynamics impacted by the pandemic in different ways^(3,37)^. In this condition, older adults stopped doing their usual activities in the community and socializing, shopping, going to church, spending time with family members, doing physical and leisure activities.

It is emphasized the importance of creating strategies for older adults, especially for those who have a higher risk of vulnerability, such as social inequalities, greater fragility in health and other conditions. Therefore, strategies or public policies should be listed for the maintenance of healthy aging in the post-COVID-19 pandemic context or in the imminence of other public emergencies, thus offering different forms of formal and informal support network, including new methodologies, such as technology insertion^(39-40)^.

### Study limitations

As a limitation, the difficult access to older adults due to social distancing is exposed, preventing all interviews from being conducted in person.

### Contributions to nursing, health, and public policies

The results contribute to nursing in the sense of enabling knowledge of older adults’ self-care perspectives in the context of a public emergency, as well as, from this, allowing reflections in order to develop strategies that contribute in these contexts to self-care in a more comprehensive way, in which healthy aging premises should still be prioritized. Contributions to public policy concern the fact of ratifying that its construction should enable access to information and popularization of science in the means of information and communication used by the population, generating reliable and understandable sources, in order to decimate false news, which can generate impacts and lethality, especially for older adults. Another contribution to public policy would be using the term “risk group” that such policies undertake and that can act against its own purpose, stigmatizing a population group.

## FINAL CONSIDERATIONS

The findings of this research showed that the self-care process of community-dwelling older adults was permeated by their experiences during the COVID-19 pandemic, whose prevention practices were influenced by different sources of information.

The results of this theoretical construction, synthesized in the phenomenon “Experienced self-care in old age during the COVID-19 pandemic”, showed that all older adults participating in the research, regardless of diagnosis of COVID-19, were impacted even if indirectly, which was reflected in preventive self-care practices influenced by the means of information about the disease and the stigma of being considered a “risk group”.

It is concluded that the experiences of older adults in pandemic scenarios and the different factors that influence the self-care process should be the target of health policies and programs aimed at older adults. It is also emphasized the need to carry out new studies in other social realities in which older adults are inserted and with other approaches that contribute to the subject studied.
